# Supplementary figures and images for: Dive behaviour and foraging effort of female Cape fur seals Arctocephalus pusillus pusillus
Source: R Soc Open Sci. 2019 Oct 16;6(10):191369. doi: 10.1098/rsos.191369 (PMC6837185; doi:10.1098/rsos.191369)

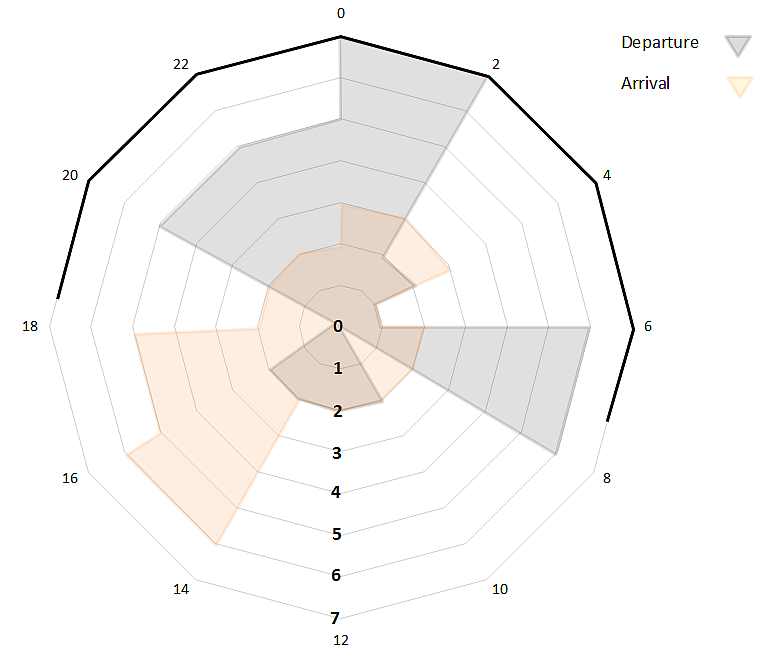

Supplement: Figure S1 [file rsos191369supp1.tif]

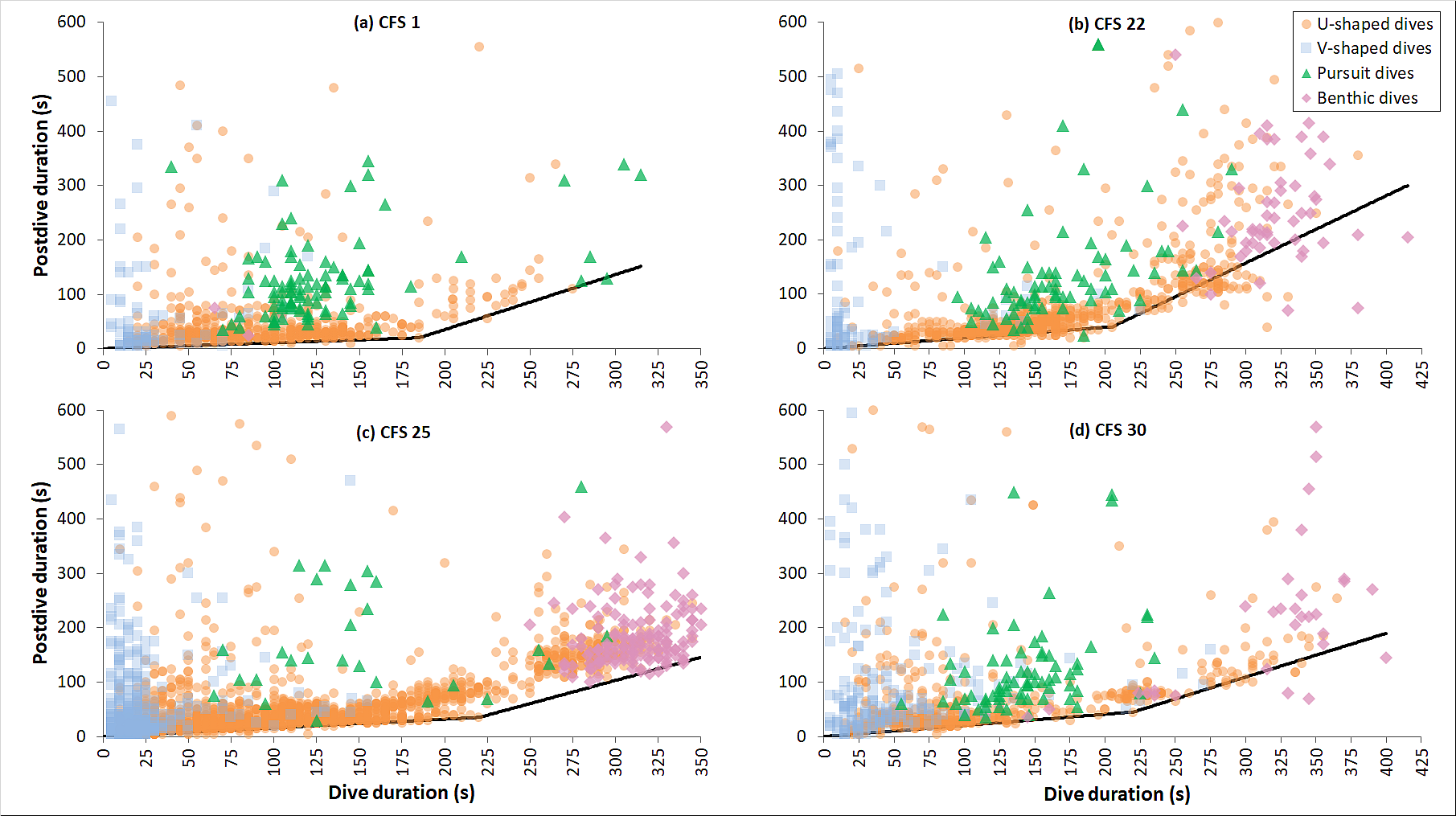

Supplement: Figure S2 [file rsos191369supp2.tif]
