# Supplementary material for: Dive behaviour and foraging effort of female Cape fur seals Arctocephalus pusillus pusillus
Source: R Soc Open Sci. 2019 Oct 16;6(10):191369. doi: 10.1098/rsos.191369 (PMC6837185; doi:10.1098/rsos.191369)
Supplement: Table S1 [file rsos191369supp3.docx]

| **Seal** | **Year** | **N** | **Dive duration (s)** | | | | | **Dive depth (m)** | | | | |
| --- | --- | --- | --- | --- | --- | --- | --- | --- | --- | --- | --- | --- |
|  |  |  | Mode | Median | Range | Mean | SE | Mode | Median | Range | Mean | SE |
| 1 | 2006 | 762 | 110 | 90 | 5–315 | 95.7 | 1.9 | 7 | 19.5 | 4.5–199 | 29.7 | 1.1 |
| 2 | 2006 | 1406 | 120 | 95 | 5–305 | 93.8 | 1.2 | 4.5 | 27.25 | 4.5–171.5 | 48.5 | 1.2 |
| 3 | 2006 | 1536 | 115 | 120 | 10–350 | 125.1 | 1.2 | 4.5 | 87.5 | 4.5–206 | 83.6 | 1.1 |
| 4 | 2006 | 460 | 115 | 120 | 5–290 | 144.9 | 3.3 | 25 | 28.25 | 4.5–149 | 64.8 | 2.5 |
| 5 | 2006 | 161 | 195 | 210 | 25–375 | 218.0 | 4.2 | 124.5 | 112 | 4.5–129 | 107.0 | 1.8 |
| 6 | 2006 | 1480 | 95 | 100 | 5–345 | 98.7 | 1.0 | 8.5 | 63 | 4.5–196 | 59.2 | 0.9 |
| 7 | 2006 | 1289 | 265 | 250 | 5–585 | 222.5 | 3.6 | 4.5 | 176.5 | 4.5–454 | 149.3 | 3.1 |
| 8 | 2006 | 1478 | 115 | 120 | 5–495 | 160.3 | 2.9 | 14 | 20 | 4.5–313.5 | 67.3 | 2.4 |
| 9 | 2006 | 1588 | 85 | 90 | 5–350 | 102.9 | 1.6 | 9 | 14 | 4.5–182.5 | 30.4 | 1.0 |
| 10 | 2006 | 2196 | 110 | 105 | 5–380 | 101.3 | 1.2 | 4.25 | 34 | 4.2–200.5 | 49.1 | 0.9 |
| 11 | 2007 | 908 | 80 | 95 | 5–405 | 129.7 | 3.2 | 13 | 28.5 | 4.1–191 | 62.0 | 2.1 |
| 12 | 2007 | 675 | 165 | 155 | 5–430 | 168.4 | 3.5 | 16.5 | 61 | 4.5–186.5 | 76.5 | 2.3 |
| 13 | 2007 | 755 | 315 | 165 | 5–380 | 190.6 | 4.1 | 162.5 | 99.5 | 4.5–278.2 | 91.9 | 2.4 |
| 14 | 2007 | 1015 | 140 | 128 | 4–364 | 143.3 | 2.4 | 4.5 | 89 | 4.5–189.5 | 86.7 | 1.7 |
| 15 | 2007 | 1288 | 110 | 110 | 5–355 | 120.1 | 2.0 | 24 | 29 | 4.1–181 | 55.3 | 1.5 |
| 16 | 2007 | 843 | 25 | 150 | 5–485 | 176.2 | 4.1 | 7.5 | 42 | 4.5–305 | 94.5 | 3.3 |
| 17 | 2007 | 1677 | 110 | 115 | 5–320 | 111.6 | 1.4 | 4.5 | 59 | 4.2–189.5 | 61.6 | 1.1 |
| 18 | 2007 | 1366 | 10 | 135 | 5–460 | 155.3 | 3.0 | 4.5 | 52 | 4.1–232 | 81.2 | 2.0 |
| 19 | 2007 | 335 | 210 | 215 | 40–261 | 215.3 | 1.3 | 127.5 | 127.5 | 12–131.5 | 124.8 | 0.7 |
| 20 | 2008 | 1095 | 10 | 80 | 5–530 | 113.3 | 3.3 | 4.5 | 24.55 | 4.1–238 | 59.5 | 2.1 |
| 21 | 2008 | 445 | 10 | 120 | 5–380 | 139.2 | 4.6 | 5 | 25.5 | 4.2–156 | 50.6 | 2.4 |
| 22 | 2008 | 991 | 145 | 145 | 5–415 | 156.3 | 2.7 | 196 | 45.75 | 4.3–202 | 75.2 | 2.1 |
| 23 | 2008 | 592 | 10 | 115 | 5–410 | 135.8 | 4.2 | 4.5 | 24.5 | 4.3–172 | 46.0 | 2.0 |
| 24 | 2008 | 1448 | 68 | 96 | 4–492 | 158.8 | 3.7 | 4.5 | 45.5 | 4.5–439 | 103.6 | 3.1 |
| 25 | 2008 | 2317 | 15 | 90 | 5–355 | 116.4 | 2.0 | 4.5 | 18 | 4.5–200 | 52.7 | 1.4 |
| 26 | 2008 | 547 | 320 | 155 | 10–375 | 191.3 | 4.3 | 175 | 102.5 | 4.5–175.5 | 85.5 | 2.7 |
| 27 | 2008 | 545 | 115 | 195 | 15–415 | 229.6 | 5.2 | 6 | 125.5 | 4.5–192 | 101.9 | 2.9 |
| 28 | 2008 | 333 | 335 | 315 | 5–420 | 243.1 | 6.8 | 195.5 | 156 | 4.5–197 | 121.0 | 4.0 |
| 29 | 2008 | 1647 | 105 | 110 | 5–430 | 109.4 | 1.7 | 5 | 25.5 | 4.5–249 | 46.4 | 1.2 |
| 30 | 2008 | 795 | 75 | 85 | 5–400 | 115.7 | 3.1 | 4.5 | 19.5 | 4.5–209.5 | 51.5 | 2.1 |
| 31 | 2008 | 556 | 20 | 130 | 5–420 | 152.7 | 4.9 | 5.5 | 34 | 4.5–185.5 | 66.3 | 2.6 |
| 32 | 2008 | 1035 | 120 | 120 | 5–330 | 132.6 | 2.3 | 167 | 89.5 | 4.5–174 | 85.7 | 1.7 |
