# Supplementary material for: Dive behaviour and foraging effort of female Cape fur seals Arctocephalus pusillus pusillus
Source: R Soc Open Sci. 2019 Oct 16;6(10):191369. doi: 10.1098/rsos.191369 (PMC6837185; doi:10.1098/rsos.191369)
Supplement: Table S2 [file rsos191369supp4.docx]

| **Model** | **df** | **LogLik** | **AICc** | **ΔAICc** | **Wt** |
| --- | --- | --- | --- | --- | --- |
| **Foraging Trip Duration ~** |  |  |  |  |  |
| Standard length + Proportion of bethic dives | 4 | -73.89 | 157.28 | 0 | 0.20 |
| Proportion of bethic dives | 3 | -75.68 | 158.22 | 0.94 | 0.13 |
| Standard length + Body condition index + Proportion of bethic dives | 5 | -73.27 | 158.85 | 1.57 | 0.09 |
| Axillary girth + Proportion of bethic dives | 4 | -74.92 | 159.32 | 2.04 | 0.07 |
| Body condition index + Proportion of bethic dives | 4 | -75.18 | 159.85 | 2.57 | 0.06 |
| **Dive rate ~** |  |  |  |  |  |
| Year | 4 | -220.67 | 450.83 | 0 | 0.11 |
| Body condition index | 3 | -222.42 | 451.69 | 0.87 | 0.07 |
| - | 2 | -223.73 | 451.87 | 1.04 | 0.06 |
| Year + Body condition index | 5 | -220.11 | 452.53 | 1.71 | 0.05 |
| Proportion of benthic dives | 3 | -222.87 | 452.59 | 1.77 | 0.04 |
| **Proportion of benthic dives ~** |  |  |  |  |  |
| Proportion of nocturnal dives | 3 | -130.51 | 267.87 | 0 | 0.38 |
| Body condition index + Proportion of nocturnal dives | 4 | -129.91 | 269.31 | 1.43 | 0.19 |
| Standard length + Proportion of nocturnal dives | 4 | -130.36 | 270.21 | 2.34 | 0.12 |
| Axillary girth + Proportion of nocturnal dives | 4 | -130.50 | 270.49 | 2.62 | 0.10 |
| Axillary girth + Body condition index + Proportion of nocturnal dives | 5 | -129.71 | 271.73 | 3.86 | 0.06 |
| **Proportion of dives exceeding bADL ~** |  |  |  |  |  |
| Proportion of benthic dives | 3 | 29.43 | -51.15 | 0 | 0.39 |
| Axillary girth + Proportion of benthic dives | 4 | 29.85 | -48.62 | 2.53 | 0.11 |
| Standard length + Proportion of benthic dives | 4 | 29.81 | -48.55 | 2.60 | 0.11 |
| Dive rate + Proportion of benthic dives | 4 | 29.64 | -48.21 | 2.94 | 0.09 |
| Body condition index + Proportion of benthic dives | 4 | 29.47 | -47.86 | 3.29 | 0.08 |
